# *BRCA1*, *BRCA2* and *PALB2* mutations and *CHEK2* c.1100delC in different South African ethnic groups diagnosed with premenopausal and/or triple negative breast cancer

**DOI:** 10.1186/s12885-015-1913-6

**Published:** 2015-11-17

**Authors:** F. Z. Francies, T. Wainstein, K. De Leeneer, A. Cairns, M. Murdoch, S. Nietz, H. Cubasch, B. Poppe, T. Van Maerken, B. Crombez, I. Coene, R. Kerr, J. P. Slabbert, A. Vral, A. Krause, A. Baeyens, K. B. M. Claes

**Affiliations:** 1 iThemba LABS-National Research Foundation, Somerset West, South Africa; 2 Department of Radiation Sciences, University of the Witwatersrand, Johannesburg, South Africa; 3 Division of Human Genetics, School of Pathology, University of the Witwatersrand, Johannesburg, South Africa; 4 Center for Medical Genetics, Ghent University Hospital, Ghent, Belgium; 5 Department of Surgery, Charlotte Maxeke Johannesburg Academic Hospital and Donald Gordon Medical Centre, Johannesburg, South Africa; 6 Batho Pele Breast Unit, Chris Hani Baragwanath Academic Hospital, Johannesburg, South Africa; 7 Department of Basic Medical Sciences, Ghent University, Ghent, Belgium; 8 Division of Human Genetics, National Health Laboratory Services, Johannesburg, South Africa

**Keywords:** Triple negative breast cancer, Premenopausal breast cancer, *BRCA* mutations, South Africa

## Abstract

**Background:**

Current knowledge of the aetiology of hereditary breast cancer in the four main South African population groups (black, coloured, Indian and white) is limited. Risk assessments in the black, coloured and Indian population groups are challenging because of restricted information regarding the underlying genetic contributions to inherited breast cancer in these populations. We focused this study on premenopausal patients (diagnosed with breast cancer before the age of 50; *n* = 78) and triple negative breast cancer (TNBC) patients (*n* = 30) from the four South African ethnic groups. The aim of this study was to determine the frequency and spectrum of germline mutations in *BRCA1*, *BRCA2* and *PALB2* and to evaluate the presence of the *CHEK2* c.1100delC allele in these patients.

**Methods:**

In total, 108 South African breast cancer patients underwent mutation screening using a Next-Generation Sequencing (NGS) approach in combination with Multiplex Ligation-dependent Probe Amplification (MLPA) to detect large rearrangements in *BRCA1* and *BRCA2*.

**Results:**

In 13 (12 %) patients a deleterious mutation in *BRCA1*/*2* was detected, three of which were novel mutations in black patients. None of the study participants was found to have an unequivocal pathogenic mutation in *PALB2*. Two (white) patients tested positive for the *CHEK2* c.1100delC mutation, however, one of these also carried a deleterious *BRCA2* mutation. Additionally, six variants of unknown clinical significance were identified (4 in *BRCA2,* 2 in *PALB2*), all in black patients. Within the group of TNBC patients, a higher mutation frequency was obtained (23.3 %; 7/30) than in the group of patients diagnosed before the age of 50 (7.7 %; 6/78).

**Conclusion:**

This study highlights the importance of evaluating germline mutations in major breast cancer genes in all of the South African population groups. This NGS study shows that mutation analysis is warranted in South African patients with triple negative and/or in premenopausal breast cancer.

**Electronic supplementary material:**

The online version of this article (doi:10.1186/s12885-015-1913-6) contains supplementary material, which is available to authorized users.

## Background

Breast cancer is the most common cancer amongst South African women with a lifetime risk of 1 in 32 [1]. South Africa is a country consisting of citizens from diverse ethnic groups. These include: black/African (79.8 %), white/Caucasian (8.7 %), mixed ancestry/coloured (9.0 %) and Indian/Asian (2.5 %) (Statistics South Africa, 2013) [2]. According to the most recent report from the National Cancer Registry of South Africa, the lifetime risk of developing breast cancer differs according to ethnicity. The lifetime risk is 1/53 in black women, 1/15 in white women, 1/21 in coloured women and 1/20 in Indian women (National Cancer registry, NHLS, 2006) [1].

Breast cancer has a strong heritable component, with approximately 15–20 % of cases exhibiting a family history of the disease [3, 4]. Mutations in genes such as *BRCA1* and *BRCA2* lead to autosomal dominant inherited cancer susceptibility and confer a high lifetime risk of breast cancer, as well as ovarian and other cancers. Recently it was suggested that the risk to develop breast cancer for *PALB2* mutation carriers is as high as the risk borne by *BRCA2* mutation carriers [5]. Identification of mutations in these genes through clinical genetic testing enables patients to undergo screening and prevention strategies, some of which provide reduced morbidity. In addition, the c.1100delC mutation in *CHEK2* has been identified as a susceptibility allele with incomplete penetrance and is associated with moderate lifetime risks of breast cancer. Data on the prevalence and spectrum of mutations in these genes are widely available for individuals of European descent. However, data for cohorts with African ancestry are scarce [6].

A few South African studies on mutations in *BRCA1*, *BRCA2* and *PALB2* are available [7–10].Three South African population groups exist in which the presence of *BRCA1/2* founder mutations occur; these are the Ashkenazi Jewish population [11], the Afrikaans population [7] and the black Xhosa population [10]. Other family-specific mutations have also been identified, as is typical of populations elsewhere. Table 1 shows data from studies done in South Africa to date. These studies have been performed mostly in white breast cancer patient cohorts. Furthermore, African populations are known to exhibit greater genomic diversity when compared to white populations, and genetic findings in one population cannot necessarily be extrapolated to another [12]. Consequently, there is a need to establish the aetiology of inherited breast cancer in this population. The epidemiology of breast cancer in South African black populations exhibits a number of unique trends when compared to other population groups worldwide. The difference in underlying genetic architecture, family structure, limited financial and human resources, limited community knowledge of breast cancer, limited information on family history and historical difficulty accessing health care, makes it more complex to perform risk assessments in these populations [13]. Overall, the cancer incidence in sub-Saharan Africa is lower as compared to developed countries but there is evidence to suggest changes in the disease burden as the impact of communicable diseases is mitigated [14]. South African women tend to be diagnosed with breast cancer at younger ages [15–17]. However, the diagnosis only occurs at advanced stage due to the lack of awareness, access to diagnostic centres available and limited screening. Hence, the inclusion criterion for a “young” breast cancer or premenopausal (PM) breast cancer patient was set at 50 years (See Additional file 1: Table S1). While this could be due to a younger population structure, it is possible that these younger women carry unique mutations in certain genes. Breast cancer in young women is correlated with aggressive tumour progression, lack of expression of receptors and poor prognosis [18]. Furthermore, it is often attributed to a genetic predisposition with germline mutations in the *BRCA1/2* genes [19–22]. Younger women of African descent are known to be in the high-risk group with decreased survival rates [23].

**Table 1:** Literature overview on *BRCA1* and *BRCA2* mutations detected in a South African population

Study (Reference)	Ethnic group	Gene	Mutation detected	Patients/families tested	Frequency (%)	Detection method
Yawitch & Van Rensburg 2000 [51]	Black	*BRCA1*	N/A	0/206	0	PTT and SSCP/HA; limited to regions with Afrikaner founder mutations
Reeves et al., 2004 [7]	White/Ashkenazi Jewish	*BRCA1*	c.68_69delAG	4/18	4.4	PTT and SSCP/HA
	White	*BRCA1*	c.329dupA	1/18	1.1	
	White	*BRCA1*	c.1008dupA	1/18	1.1	
	White	*BRCA1*	c.1352C > A; p.S451*	1/18	1.1	
	White/Afrikaner	*BRCA1*	c.1374delC	2/18	2.2	
	White/Afrikaner	*BRCA1*	c.2641G > T; p.E881*	5/18	5.6	
	Indian	*BRCA1*	c.4957insC	1/18	1.1	
	White/Ashkenazi Jewish	*BRCA1*	c.5266dupC	3/18	3.3	
Schlebusch et al., 2010 [52]	White/Afrikaner, Ashkenazi Jewish, Black, Indian	*BRCA1*	N/A	26/129	20.2	PTT and SSCP/HA and MLPA
		*BRCA2*	N/A	43/129	33.3	
Sluiter et al., 2011 [9]	White/Afrikaner	*BRCA1 + BRCA2*	N/A	0/36		MLPA
	White/Ashkenazi Jewish	*BRCA1*	Ex23-24del	1/30	3.3	
		*BRCA2*	N/A	0/30		
Van der Merwe et al., 2012 [10]	Coloured	*BRCA1*	c. 1504_1508delTTAAA	1/105	1.0	PTT and SSCP/HA
		*BRCA1*	c. 2641G > T;p. E881*	1/105	1.0	
		*BRCA2*	c. 2826_2829delAATT	1/105	1.0	
		*BRCA2*	c. 5771_5774delTTCA	4/105	3.8	
		*BRCA2*	c. 6448dupTA	1/105	1.0	
		*BRCA2*	c. 7934delG	1/105	1.0	
	Black	*BRCA2*	c. 5771_5774delTTCA	4/16	25.0	
Schoeman et al., 2013 [13]	White, Mixed Ancestry, Black	*BRCA1*	c. 2641G > T; p. E881*	7/302	2.3	SSCP/HA
		*BRCA1*	c. 68_69delAG	2/302	0.7	
		*BRCA1*	c. 1374delC	2/302	0.7	
		*BRCA1*	c. 5266dupC	1/302	0.3	
		*BRCA2*	c. 7934delG	17/302	5.6	
		*BRCA2*	c. 5771_5774delTTCA	7/302	2.3	
		*BRCA1*	N/A	4/302	1.3	PTT
		*BRCA2*	N/A	5/302	1.7	
		*BRCA1*	N/A	2/302	0.7	Sequencing
		*BRCA2*	N/A	2/302	0.7	Sequencing
		*BRCA1*	N/A	18/302	6.0	

*PTT* protein truncation test, *SSCP/HA* PCR-single strand conformation polymorphism/heteroduplex analysis, *N/A* mutations were not described; * indicates the presence of a premature stop codon (cfr. nomenclature HGVS (Human Genome Variation Society))

Another factor that is generally considered as an indicator of genetic susceptibility to breast cancer is the so-called “triple negative” histological phenotype. Approximately 15 % of breast cancers lack the expression of estrogen receptors, progesterone receptors and HER2/NEU receptors and are known as triple negative breast cancer (TNBC) [24]. This type of breast cancer is associated with an aggressive disease progression, higher histological grade, poor prognosis, high rate of recurrence and decreased survival rates. The frequent occurrence of TNBC is strongly correlated with younger patients of African descent and increased incidence has been noted among black South African breast cancer patients [16, 17, 25]. The strong association between TNBC and mutations in the *BRCA1* gene, seen in European and American populations [26, 27], has not been investigated in a South African cohort.

This study aimed to evaluate the contribution of germline *BRCA1*, *BRCA2* and *PALB2* mutations and the *CHEK2* c.1100delC allele to breast cancer in a high-risk South African cohort. Individuals included in the study were of different ethnicities (with a majority from the understudied black population) and had been diagnosed with premenopausal breast cancer (less than 50 years) or exhibited the “triple negative” histological phenotype. We chose to analyse *BRCA1*, *BRCA2* and *PALB2* as associated risks are well established and clinically relevant. In addition, the prevalence of *CHEK2* c.1100delC was evaluated in this cohort and compared with the prevalence in individuals of European ancestry. We applied a cost efficient next generation sequencing (NGS) approach for analysis of the complete coding regions of *BRCA1*, *BRCA2* and *PALB2* [28]. Furthermore, large rearrangements have been reported in both *BRCA1* and *BRCA2* in several populations which may be missed by sequencing. We therefore complemented the sequencing approach with multiplex ligation-dependent probe amplification (MLPA), for these two genes.

## Methods

### Patients

EDTA blood samples of 108 breast cancer patients were collected from breast clinics in two state hospitals and a private hospital in Johannesburg - Charlotte Maxeke Johannesburg Academic Hospital, Chris Hani Baragwanath Academic Hospital and Wits Donald Gordon Medical Centre respectively. Patients were selected if their tumour was triple-negative (TN), and/or their breast cancer diagnosis was premenopausal. All patients were categorized as black, white, Indian or coloured based on patients’ self-reported data from questionnaires. The cohort consisted of 85 black patients (78.7 %), 16 white patients (14.8 %), 5 Indians (4.6 %) and 2 coloureds (1.9 %). Table 2 presents the overview of the distribution of ethnicity in the cohort. All patients signed informed consent. Pathology data were obtained from the hospital files. Genetic counselling was offered to the patients, prior to obtaining their consent.

**Table 2:** Overview of distribution of ethnicity in our South African cohort

		Black (%)	White (%)	Indian (%)	Coloured (%)
Dx < 50 *n* = 92	TNBC	7 (7.6)	4 (4.3)	2 (2.2)	1 (1.1)
	Not TNBC	70 (76.1)	5 (5.4)	2 (2.2)	1 (1.1)
Dx > 50 *n* = 16	TNBC	8 (50.0)	7 (43.8)	1 (6.3)	0
Total *n* = 108		85 (78.7)	16 (14.8)	5 (4.6)	2 (1.9)

Dx: Age at diagnosis

The study was approved by the Human Research Ethics Committee (Medical), University of the Witwatersrand (No. M091023; M110922; M130450).

### DNA extraction

Genomic DNA was extracted from 4 - 6 ml of peripheral blood using a modified version of the standard salting out method [29].

### Target enrichment, library preparation and sequencing

*BRCA1*, *BRCA2* and *PALB2* analysis was successfully conducted on 108 samples using Illumina’s Miseq desktop sequencer. Target enrichment was achieved by high throughput PCR. Primers were designed for the complete coding region including splice site regions of *BRCA1* (31 amplicons), *BRCA2* (42 amplicons) and *PALB2* (19 amplicons) using Primer XL (www.pxlence.com). PCR conditions according to the protocol described by De Leeneer et al. were utilised [28].

Library preparation was performed using a modified version of the Nextera XT (Illumina) protocol. Sequencing was conducted on the MiSeq v2 instrument (Illumina Inc.) according to manufacturer’s instructions. The approach is described in detail by De Leeneer et al. [28].

### Sanger sequencing

All genetic variants and pathogenic mutations identified via NGS were confirmed with Sanger sequencing. For confirmation by Sanger sequencing, an independent PCR amplification step was performed. In addition, the presence of all deleterious mutations was confirmed on an independently extracted DNA sample. All fragments with a coverage of <28× were also analysed by Sanger sequencing. For an overview of the number of amplicons that required Sanger sequencing, refer to Additional file 2: Table S2.

Nucleotide positions and protein translation correspond to reference sequence and Genbank account number NM_007294.3; NP_009225.1 for *BRCA1*, NM_000059.3; NP_000050.2 for *BRCA2*, NM_024675.3; NP_078951.2 for *PALB2* and NM_007194.3 for *CHEK2* c.1100delC. Nucleotide numbering uses the A of the ATG translation initiation start site as nucleotide 1.

### MLPA

Large genomic rearrangements and/or gene dosage alterations in both the *BRCA1* and *BRCA2* genes were screened for in 108 patient samples using MLPA. *BRCA1* MLPA analysis was performed using the SALSA MLPA P002 probemix (version C2-1113) (MRC-Holland) and *BRCA2*/*CHEK2* MLPA using the SALSA MLPA P045 probemix (version B3-1113) (MRC-Holland). MLPA setup was performed according to the manufacturer’s protocol. Fragment detection and sizing was conducted using capillary gel electrophoresis on the ABI 3730XL genetic analyser (Applied Biosciences). All fragments positive for the *CHEK2* mutation (c.1100delC) in the MLPA analysis were confirmed with Sanger sequencing.

The screening was performed in a research setting. We used the infrastructure and the protocols supplied by a molecular diagnostic laboratory with an ISO15189 accreditation.

### Data analysis

Mapping of sequencing data was performed with CLC bio Genomics Workbench v6 software (CLC bio Inc.). Various in-house scripts were used for sequence analysis [28]. The Sanger sequencing data were analysed using SeqPilot v4.1.2 build 512 and SeqSpace v2.5.0. MLPA data were analysed using Coffalyser (MRC-Holland).

Variants of unknown significance (VUS) were evaluated using *in silico* mutation interpretation software – Alamut. We used the computational algorithms of SIFT, AlignGVGD, Polyphen and Mutation Taster for missense varaints and the splice site prediction programs SpliceSiteFinder, MaxEntScan, NNSPLICE, GeneSplicer and Human Splicing Finder for intronic, silent and missense variants. Based on these predictions and in combination with a study of the literature and published minor allele frequencies, variants were classified in five classes. Unfortunately, due to limited availability of data, Bayesian likelihood analyses could not be performed to calculate the degree of likelihood of pathogenicity. Therefore, we applied the following rules:

- Variants with a MAF (minor allele frequency) of  > 0.01 were classified as class 1 (data not shown)
- Variants were classified as class 2 if all prediction programs provided neutral scores (data not shown)
- Variants with two or more programs with deleterious predictions were allocated to class 3 (Table 5)
- All truncating and unequivocal splice site variants were considered as deleterious, in addition to missense variants in the RING domain of BRCA1 (class 4–5) (Table 3)

**Table 3:** *BRCA1, BRCA2* and *CHEK2* germline pathogenic mutations identified in triple negative and premenopausal breast cancer patients using NGS and MLPA

Patient no.	Ethnicity	Category	Gene	Exon	Nucleotide change	Amino acid change	Mutation effect	Reference
1	White	TNBC/PM	*BRCA1*	4	c.181 T > G	p.Cys61Gly	Missense	[53]
2	Black	TNBC/PM	*BRCA1*	4	c.212G > A	p.Arg71Lys	Missense	[54]
3	Indian	TNBC/PM	*BRCA1*	10	c.3593 T > A	p.Leu1198*	Nonsense	[55]
4	Black	PM	*BRCA1*	10	c.1155G > A	p.Trp385*	Nonsense	Novel
5	Black	PM	*BRCA1*	10	c.1953_1954insA	p.Lys652fs	Frameshift	Novel
6	White	TNBC	*BRCA1* ^a^	1–2	-	-	Deletion	[30]
7	Black	PM	*BRCA2*	7	c.582G > A	p.Trp194*	Nonsense	Novel
8	Black	TNBC	*BRCA2*	11	c.5771_5774delTTCA	p.Ile1924fs	Frameshift	[10]
9	White	PM	*BRCA2*	11	c.5213_5216delCTTA	p.Thr1738fs	Frameshift	[56]
			*CHEK2* ^a^	11	c.1100delC	p.Thr367fs	Frameshift	[39]
10	White	TNBC	*BRCA2*	17	c.7934delG	p.Arg2645fs	Frameshift	[10]
11	White	PM	*BRCA2*	17	c.7934delG	p.Arg2645fs	Frameshift	[10]
12	Indian	TNBC/PM	*BRCA2*	21	c.8754 + 1G > A	Non-coding	Splice site	[57]
13	Black	PM	*BRCA2*	23	c.9097_9098insA	p.Thr3033fs	Frameshift	[53]
14	White	PM	*CHEK2* ^a^	11	c.1100delC	p.Thr367fs	Frameshift	[39]

*PM* Premenopausal

^a^MLPA results

^*^indicates the presence of a premature stop codon (cfr. nomenclature HGVS (Human Genome Variation Society))

### Statistical analysis

Mutation frequency was calculated with 95 % confidence intervals. The Fisher’s exact test was used to compare mutation frequencies in the different groups of patients. Statistical analysis was performed with Graphpad Prism software.

## Results

In the total study population (*n* = 108), 15 heterozygous pathogenic mutations in 14 patients were identified (12.9 %; 95 % CI = 7.3–20.8 %): six in *BRCA1,* seven in *BRCA2*; two patients were found to carry *CHEK2* c.1100delC of which one patient also harboured a deleterious *BRCA2* mutation. All mutations were identified by sequencing on Miseq, except a large deletion in *BRCA1* and the *CHEK2* c.1100delC mutation which were detected by MLPA. No unequivocal deleterious mutations were identified in the *PALB2* gene (Table 3).

The distribution of *BRCA1/2* mutations among the different subgroups (TNBC and/or PM) and based on ethnicity is presented in Table 4. A significantly higher mutation detection ratio was obtained within the group of TNBC patients (7/30; 23.3 %; 95 % CI = 9.9–42.3 %) compared to the premenopausal breast cancer group without TNBC (6/78; 7.7 %; 95 % CI = 2.9–16.0 %) (*p* = 0.0432). Not surprisingly, the highest mutation detection ratio was obtained within the subgroup of TNBC patients diagnosed before the age of 50 (5/14; 35.7 %; 95 % CI = 12.7–64.9 %).

**Table 4:** *BRCA1* and *BRCA2* germline pathogenic mutations identified using NGS and MLPA in a South African cohort divided according to premenopausal diagnosis, triple negative status and ethnicity

Total *n* = 108	Dx < 50 *n* = 92 (85.2 %)				Dx > 50 *n* = 16 (14.8 %)		Total no. of mutations per ethnic group
	TNBC *n* = 14 (13.0 %)		Not TNBC *n* = 78 (72.2 %)		TNBC		
Black *n* = 85 (78.7 %)	*n* = 7		*n* = 70		*n* = 8		6 (7.1 %)
Mutations	BRCA1	BRCA2	BRCA1	BRCA2	BRCA1	BRCA2	
	c.212G > A	-	c.1155G > A	c.582G > A	-	c.5771_5774delTTCA	
	-	-	c.1953_1954insA	c.9097_9098insA	-	-	
White *n* = 16 (14.8 %)	*n* = 4		*n* = 5		*n* = 7		5 (31.3 %)
Mutations	BRCA1	BRCA2	BRCA1	BRCA2	BRCA1	BRCA2	
	c.181 T > G	c.7934delG	-	c.7934delG	Exon 1a-2 del	-	
	-	-	-	c.5213_5216delCTTA	-	-	
Indian *n* = 5 (4.6 %)	*n* = 2		*n* = 2		*n* = 1		2 (40.0 %)
Mutations	BRCA1	BRCA2	BRCA1	BRCA2	BRCA1	BRCA2	
	c.3593 T > A	c.8754 + 1G > A	-	-	-	-	
Coloured *n* = 2 (1.9 %)	*n* = 1		*n* = 1		0		0
Mutations	-		-		-		
Total mutations per subgroup	5 (35.7 %)		6 (7.7 %)		2 (12.5 %)		

The *BRCA2* c.7934delG Afrikaner founder mutation was identified in 2 (white) patients, one with TNBC and one diagnosed with premenopausal breast cancer. In the black patient population, two previously unreported mutations were identified in *BRCA1* (c.1155G > A and c.1953_1954insA) and one in *BRCA2* (c.582G > A) (see Table 3). Six (6/85; 7.1 %; 95 % CI = 2.6–14.7 %) pathogenic *BRCA1/2* mutations were observed in the black population group and five (5/16; 31.3 %; 95 % CI = 11.0–58.7 %) in the white population group. Two mutations were identified in the Indian group (2/5; 40 %; 95 % CI = 5.3–85.3 %) and no mutations were identified either in *BRCA1* or *BRCA2* in the two coloured individuals studied.

To detect large genomic rearrangements in *BRCA1* and *BRCA2*, 108 samples were analysed using MLPA. A white TNBC patient was found to be heterozygous for a *BRCA1* exon 1a-2 deletion. Several deletions including these exons but with different breakpoints have previously been described (for an overview of deletions affecting these exons: [30]). As the number of large rearrangements reported in *PALB2* is extremely small [31], MLPA for *PALB2* was not conducted in this cohort.

The *CHEK2* mutation (c.1100delC) was observed in 2/108 (1.9 %) patients. Both of these patients were white, premenopausal patients. One of these patients was also positive for a deleterious *BRCA2* mutation.

In addition to pathogenic mutations, several VUS were identified: 1 in *BRCA1*, 3 in *BRCA2* and 2 in *PALB2*. In Table 5 we provide an overview of the variants which were classified as class 3 based on *in silico* prediction programs. Three of the four *in silico* prediction programs used classified the *BRCA2* variant c.9875C > T and c.7712A > G as “probably damaging”. The *BRCA2* variant c.9875C > T was identified in two black patients. Two of the four prediction programs consulted classified the *PALB2* variants c.118A > G and c.2845 T > C as “probably damaging”.

**Table 5:** *In silico* predictions obtained for variants of unknown significance in the South African cohort

					*In silico* prediction programs					
Ethnicity	Variant	Gene	Amino acid change	Occurrence	Classification	Align GVGD^a^	SIFT	Mutation Taster	PolyPhen	Refs
Black	c.1843_1845delTCT	*BRCA1*	p.Ser615del	1	3	-	-	-	-	[58–60]
Black	c.4798_4800delAAT	*BRCA2*	p.Asn1600del	1	3	-	-	-	-	[61]
Black	c.7712A > G	*BRCA2*	p.Glu2571Gly	1	3	C0	Deleterious	Disease causing	Probably damaging	[62]
Black	c.9875C > T	*BRCA2*	p.Pro3292Leu	2	3	C0	Affect protein function	Disease causing	Probably damaging	[63]
Black	c.118A > G	*PALB2*	p.Arg40Gly	1	3	C0	Affect protein function	Polymorphism	Probably damaging	Novel
Black	c.2845 T > C	*PALB2*	p.Cys949Arg	1	3	C0	Affect protein function	Disease causing	Probably damaging	Novel

^a^Spectrum of prediction classes (C0, C15, C25, C35, C45, C55, C65) with C0 less likely to be deleterious and C65 most likely

## Discussion

The current study is the first study performing mutation analyses in *BRCA1, BRCA2* and *PALB2* and determining the frequency of *CHEK2* c.1100delC in triple negative and/or premenopausal breast cancer patients in South Africa through both next generation sequencing and large rearrangement testing. In total we detected 13 *BRCA1/2* mutations in our study cohort of 108 patients (12 %; 95 % CI = 6.6–19.7 %), thus reinforcing the important contribution of germline *BRCA1* and *BRCA2* mutations to inherited breast cancer in this mixed South Africa cohort. Two patients harboured a *CHEK2* c.1100delC mutation, one of them in combination with a deleterious *BRCA2* mutation. Previous studies done on South African breast cancer populations reported *BRCA1/2* mutation frequenciess of 1 to 25 % [7–10] (for an overview: see Table 1). The prevalence of mutations in *BRCA1/2* genes in these South African studies varies by inclusion criteria, ethnicity and mutation screening techniques used. None of these studies looked specifically at TNBC or premenopausal patients.

The mutation frequency was higher in the subgroup of TNBC than in the premenopausal breast cancer patients: 23.3 % (7/30) of TNBC patients harbour a pathogenic mutation in either *BRCA1* or *BRCA2,* compared to 12.0 % (11/92) of all premenopausal breast cancer patients.

Various studies have shown the frequency of *BRCA1* mutations to be higher than *BRCA2* in patients exhibiting the triple negative phenotype [27, 32, 33]. In our study 13.3 % (4/30) of TNBC patients had a pathogenic mutation in *BRCA1* compared to 10 % (3/30) in *BRCA2*.

In our premenopausal cohort, the prevalence of *BRCA1* mutations were similar (5/92; 5.4 %) to *BRCA2* mutations (6/92; 6.5 %). *BRCA2* mutations are in general less frequent than *BRCA1* in younger white women with breast cancer [19]. A relatively high number of *BRCA2* mutations compared to *BRCA1* has been reported in other studies of young black populations [34–36] and is contradictory to the scenario in Western populations. This could be due to the unique genetic background of African patients.

In the black population, the overall frequency of mutations identified was 7.1 % as compared to 31.3 % in the white population. Due to the presence of the *BRCA2* c.7934delG Afrikaner founder mutation, *BRCA2* is the most important contributor in the white population in our study cohort, while *BRCA1* and *BRCA2* mutations were observed in equal numbers in the black patients studied. We identified neither the Ashkenazi Jewish nor the Xhosa mutations in our study groups. Our patient cohort was recruited in the region of Johannesburg and is characterized by diverse population structure/ethnic backgrounds. Therefore we did not anticipate finding a large number of founder mutations.

The *CHEK2* c.1100delC allele contributes to a moderate increased breast cancer risk. The frequency is estimated to be only 1 % in familial breast cancer and 0.5 % in early onset breast cancer [37, 38]. In the Dutch population the prevalence in the general population is 1.1 %, 2.5 % in unselected breast cancer cases, and up to 4.9 % in familial breast cancer cases [39]. Within our South African cohort we identified this allele in two white patients (2/16 = 12.5 %), but in none of the patients from other ethnicities (0/92). White Afrikaner South Africans mainly descend from Dutch immigrants which could explain the higher percentage of *CHEK2* c.1100delC in this cohort.

Previous studies that aimed to clarify the prevalence of *BRCA1/2* mutations in black populations from other parts of Africa and African Americans have indicated similar rates [6, 22, 27, 36, 40]; although it is difficult to compare them since eligibility criteria for study participation varies extensively. Churpek et al. [40] reported a pick-up rate of 26 % (47/180) for pathogenic mutations in a group of black patients with early onset disease (age of diagnosis <45) and 25 % pick-up rate (26/103) for pathogenic mutations in triple negative black patients. Here we report *BRCA1/2* mutation frequency of 14 % (1/7) in the premenopausal triple negative black subgroup. Our overall mutation detection rate of *BRCA1/2* mutations in the black premenopausal breast cancer patients was 6.5 % (5/77). This is similar to the mutation rate reported in a study by Pal et al. [22] in young black African American breast cancer patients (9 %; 13/144). Although the prevalences are similar among the studies on West African, African American breast cancers and our study, we identified 3 novel mutations in the South African black patients. Furthermore, historical evidence has shown that African Americans descend from West African ancestry and so it is not surprising that there are some differences between these two and the South African black population, who have some distinct genetic differences at the population level [12, 41].

Large genomic rearrangements in *BRCA*, detected with MLPA, were only observed in 0.9 % (1/108) of our cohort. No large rearrangements were identified in the black South African breast cancer patients. Generally, low frequencies for large rearrangements have been reported in black patients, e.g. Pal et al., [22], detected 2 rearrangements in 144 young African-American women with breast cancer (1.4 %), both of which were in *BRCA1*. Zhang et al., [42] reported one *BRCA1* exon deletion (0.3 %) in a cohort of 352 Nigerian breast cancer patients. In another South African study on 52 unrelated families of European ancestry, only 1 large deletion was detected in *BRCA1* [9]. The lack of detection in *BRCA2* led the authors to suggest that large rearrangements in *BRCA2* might not play a role in inherited breast cancer in South African patients [9]. However, to draw final conclusions on the presence of large rearrangements in both white and black South African breast cancer patients, a larger patient population should be extensively studied.

Gene sequencing techniques also resulted in the identification of several VUS. Based on *in silico* predictions, we assigned a class (class 1– 3) to each VUS for clinical interpretation [43]. VUS with a probability of increased pathogenicity are assigned a higher class. A number of studies have presented models and performed functional assays for the classification of VUS in *BRCA1*/*2* [43–46]. We detected six VUS in the 85 black patients of our cohort and none in the 16 white patients. Also other studies suggested that the frequency of VUS is higher in patients of African descent, for instance Nanda et al. [47].

A previous study conducted in a South African cohort revealed a pathogenic *PALB2* mutation in 2 % of early onset white breast cancer patients [8]. Our cohort consisted of a small number of white patients and no unequivocal deleterious mutations in *PALB2* were identified. However two missense variants with suggestive *in silico* predictions were identified (Table 5) that warrant further functional analyses. Until recently, the pathogenic effect of *PALB2* missense variants has not been firmly proven. For some missense variants in the WD40 domain (from amino acids 853–1186) [48] altered patterns of direct binding to the RAD51C, RAD51 and BRCA2 h proteins in biochemical assays have been shown [49]. We identified a missense variant in the WD40 domain (c.2845 T > C; p.Cys949Arg). In order to elucidate the pathogenicity of missense variants in *PALB2*, additional (functional, segregation) analyses are required.

We focused on identifying mutations in *BRCA1*, *BRCA2* and *PALB2* and the *CHEK2* c.1100delC mutation, as the risks for the development of breast and associated cancers with these genes have been determined by analysing large study populations. The search for the remaining genetic contribution towards breast and ovarian cancer has been carried out extensively, with numerous other genes being identified. However, at this time, the contribution and associated risks of mutations in most of these genes is not yet well established. As the prevalence of mutations in each of these genes is much lower than germline *BRCA1/2* mutations in the large cohorts (white American) of patients investigated up until now [50], international collaborations in populations of different ethnicities will be required to gain insight into the exact risks associated with mutations in these genes.

## Conclusion

This study is the first to evaluate the use of NGS technology as a diagnostic testing platform for inherited breast cancer in a South African cohort. The results presented herein are particularly relevant for inherited cancer testing in the black population of South Africa, a previously under-researched group. The NGS approach applied [28] is a cost and time effective approach; it shows great promise for *BRCA1/2* screening in developing countries like South Africa. The advent of NGS allows the costs of mutation analysis to fall dramatically, which should allow testing to become more widely available, especially in countries with limited healthcare resources, like South Africa. This will create opportunities to improve patient treatment and challenges for breast cancer multidisciplinary teams. The finding of a germline deleterious mutation could alter treatment decisions; for instance, women with germline mutations might opt for more radical surgery or may consider prophylactic surgery to the contralateral breast or ovaries.

Our results have highlighted the contribution of *BRCA1/2* germline mutations in South African breast cancer patients with triple negative breast tumours and/or premenopausal breast cancer of different ethnicities.

## Additional files

Additional file 1: Table S1. Overview of grading and staging of breast cancer on diagnosis (DOC 30 kb)
Additional file 2: Table S2. Overview of sequencing coverage per run (DOC 29 kb)

## Abbreviations

MAF: Minor allele frequency
MLPA: Multiplex ligation-dependent probe amplification
NGS: Next-generation sequencing
PM: Premenopausal
TN: Triple negative
TNBC: Triple negative breast cancer
VUS: Variants of unknown significance
