# Supplementary material for: BRCA1, BRCA2 and PALB2 mutations and CHEK2 c.1100delC in different South African ethnic groups diagnosed with premenopausal and/or triple negative breast cancer
Source: BMC Cancer. 2015 Nov 17;15:912. doi: 10.1186/s12885-015-1913-6 (PMC4647511; doi:10.1186/s12885-015-1913-6)
Supplement: Additional file 1: Table S1. — Overview of grading and staging of breast cancer on diagnosis (DOC 30 kb) [file 12885_2015_1913_MOESM1_ESM.doc]

**Supplementary Table 1: Overview of grading and staging of breast cancer on diagnosis**

| **Age** | *Average* | 45 |
| --- | --- | --- |
|  | *Median* | 42 |
|  | *Range* | 25 – 77 |
| **Grade** | *Grade 1* | 7% |
|  | *Grade 2* | 51% |
|  | *Grade 3* | 42% |
| **Stage** | *Stage I* | 9% |
|  | *Stage II* | 55% |
|  | *Stage III* | 32% |
|  | *Stage IV* | 2% |
