# Supplementary material for: BRCA1, BRCA2 and PALB2 mutations and CHEK2 c.1100delC in different South African ethnic groups diagnosed with premenopausal and/or triple negative breast cancer
Source: BMC Cancer. 2015 Nov 17;15:912. doi: 10.1186/s12885-015-1913-6 (PMC4647511; doi:10.1186/s12885-015-1913-6)
Supplement: Additional file 2: Table S2. — Overview of sequencing coverage per run (DOC 29 kb) [file 12885_2015_1913_MOESM2_ESM.doc]

**Supplementary Table 2:** Overview of sequencing coverage per run

| **Run** | **Total no. of amplicons sequenced** | **No. of amplicons with coverage <28x** | **No. of amplicons with coverage <5x** | **Average per run** | **Median per run** | **Range per run** |
| --- | --- | --- | --- | --- | --- | --- |
| 1 | 4416 | 177 (4%) | 123 (3%) | 313 | 238 | 0-7000 |
| 2 | 1248 | 38 (3%) | 25 (2%) | 208 | 185 | 0-1000 |
| 3 | 2400 | 23 (1%) | 20 (1%) | 509 | 441 | 0-2500 |
